# Non-Local SVD Denoising of MRI Based on Sparse Representations

**DOI:** 10.3390/s20051536

**Published:** 2020-03-10

**Authors:** Nallig Leal, Eduardo Zurek, Esmeide Leal

**Affiliations:** 1 Department of Systems Engineering, Universidad del Norte, Barranquilla 080001, Colombia; ezurek@uninorte.edu.co; 2 Independent Consultant, Barranquilla 080001, Colombia; esmeide0226@yahoo.com

**Keywords:** dictionary learning, image denoising, MR Images, non-local filtering, singular value decomposition, sparse representations

## Abstract

Magnetic Resonance (MR) Imaging is a diagnostic technique that produces noisy images, which must be filtered before processing to prevent diagnostic errors. However, filtering the noise while keeping fine details is a difficult task. This paper presents a method, based on sparse representations and singular value decomposition (SVD), for non-locally denoising MR images. The proposed method prevents blurring, artifacts, and residual noise. Our method is composed of three stages. The first stage divides the image into sub-volumes, to obtain its sparse representation, by using the KSVD algorithm. Then, the global influence of the dictionary atoms is computed to upgrade the dictionary and obtain a better reconstruction of the sub-volumes. In the second stage, based on the sparse representation, the noise-free sub-volume is estimated using a non-local approach and SVD. The noise-free voxel is reconstructed by aggregating the overlapped voxels according to the rarity of the sub-volumes it belongs, which is computed from the global influence of the atoms. The third stage repeats the process using a different sub-volume size for producing a new filtered image, which is averaged with the previously filtered images. The results provided show that our method outperforms several state-of-the-art methods in both simulated and real data.

## 1. Introduction

MR Imaging is a useful diagnostic technique that produces high-resolution images that are affected by different sources of quality deterioration due to the inherent limitations in the hardware, scanning time, movements of patients, and, one of the most important, noise. The presence of noise is not only a visual problem but also may interfere during the analysis and segmentation tasks [1].

A study of errors in radiology conducted by Donald L. Renfrew [2] found that errors usually involved limitations in imaging technique and acquisition of inaccurate or incomplete clinical history, among others. Previous research found that 69.23% were perceptual errors, and 6.4% of errors were due to poor image quality [3]. This research suggests the importance of guaranteeing the image quality in order to obtain better results in image segmentation and analysis for preventing errors. Despite the advance in MR imaging acquisition technology, which allowed the increment in the signal-to-noise ratio, the acquisition speed, and the resolution, MR images are still affected by noise [4], therefore the filtering of MR images continues being an active research area [5,6,7,8,9,10].

The raw captured data of an MR image are complex-valued, i.e., the image can be decomposed in amplitude and phase or real and imaginary images [11]. Both real and imaginary images are affected by additive Gaussian noise. However, magnitude MR images, which are obtained by computing the magnitude from the real and imaginary images, through a nonlinear mapping, are affected by noise that does not follow a Gaussian distribution; instead, it follows a Rician distribution.

According to Foi [12], Rician distribution has two parameters, the noise-free magnitude of the data, which is unknown, and the standard deviation of the noise that affects the real and imaginary images. Estimating the magnitude is a difficult task, because the standard deviation of Rician noise in magnitude MR images depends on the magnitude of the data itself, and the expectation of the noisy magnitude differs from the noise-free magnitude by a nonlinear function of the noise standard-deviation and of the noise-free magnitude. Foi [12] proposed a method, called Variance-Stabilization Transformation (VST), which enables methods designed for filtering additive Gaussian noise, to deal with MR images.

Some works focused on improving the MR imaging results during the scanning process [13,14]. However, when no previous treatment has been performed, we must deal with issues in the captured image. Hence, many previous works proposed methods for denoising MR images, most of them are adaptations of approaches for filtering 2D images, such as the widely known Non-Local Means (NLM) filtering, proposed by Buades et al. [15] in 2005. This method was the first approach in considering the self-similarity of the image for filtering noise. This method computes the pixel value as a non-local weighted average of all pixels in the image; the weights depend on the similarity between the patches around the pixels. This method is the basis of many proposed variants; however, blurring and blocking artifacts still affect the denoised image [16].

Coupé et al. [17] and Manjón et al. [18] introduced the first adaptations of the NLM for denoising MR Images in 2008. The proposal of Coupé et al. [17] focused on an efficient implementation that reduces the dependency on parameters by automatically tuning the smoothing parameter and selecting the most relevant voxels. Similarly, the proposal of Manjón et al. [18] focused on finding the optimal parameters for denoising MR magnitude images.

Due to the high computational complexity of NLM-based methods, some works focused on efficient implementations for processing MR images. Hu et al. [19] proposed a variant called SNLM. The proposed algorithm drastically reduces the running time to 1/20 of other NLM’s variants by randomly selecting a small subset of voxels. They introduced an optimal sampling pattern for further improvement. However, the sampling ratio needs to be high for the denoising results to be comparable to NLM. Klosowski and Frahm [20] presented an efficient implementation for real-time MR image processing, which improves details preservation. The improvement was carried out by introducing a simple weighting function based on a compact support kernel. The reported results are comparable to the block-matching three-dimensional filter. Manjón proposed to use NLM along with sparseness [21], and NLM along with sparseness plus principal components analysis (PCA) [10] for denoising MR images. The last method estimates the noise level of the image, which is assumed as white noise and filters it using non-local PCA. However, this is not satisfied when using Fourier or compressed sensing; therefore, the method cannot be applied when this assumption is not met.

The SVD is also a widely recognized technique that has been used for image denoising [22,23,24]. Rajwade et al. [25] successfully used the higher order singular value decomposition (HOSVD) for filtering natural images, although this technique require complex criteria, which involve solving optimization models, to establish the low rank approximation for filtering noise. In addition, the HOSVD suffers from artifacts when applies on homogeneous regions [26]. The work of Rawade was adapted for Zhang et al. [27] for filtering MR images. This adaptation improved the HOSVD, by adding an additional recursive stage which in general terms, repeats the HOSVD for filtering the noisy image resulting from the partial re-incorporation of the residual noise into the denoised image produced by the first stage. Zhang et al. [26] also adapted the HOSVD for filtering diffusion-weighted images. Several other works used the SVD a long with NLM for filtering MR images [28] and natural images [29,30]

Dabov et al. [31] proposed one of the most important methods in state-of-the-art image denoising, “Image denoising with block-matching and 3D filtering” (BM3D). This method exploits the self-similarity in conjunction with filtering in the transform domain, and has a variant for direct processing of volumetric data, denominated BM4D [32], which is still considered a state-of-the-art algorithm [33]. Like the BM3D, the BM4D in the first stage applies thresholding for gathering similar cubes in a 4D data structure and removes noise through a 4D transform. The second stage restores the details by using collaborative filtering. The stacked 3D cube voxels in the 4D structure lead to difficulties in removing noise in the beginning and at the end of the bands of hyperspectral image [33]. In addition, when the noise level is high, the block-matching distance does not allow the program to obtain enough similar blocks; therefore, the denoising reduces its performance, and some artifacts appear.

Another important approach that attracted attention in image processing is sparse representation. It has been used for image labeling [34], image segmentation and classification [35], image inpainting [36] and image denoising [37,38,39], among others. This approach was also used for denoising MR images. Recently, Yuan [40] proposed an efficient implementation, based on an improved Split-Bregman algorithm, for filtering MR images using a sparse tensor model. Zhang et al. [41] use clustering to gather similar patches and filter them using a sparse model, which uses sparse gradient priors to exploit the image low-rank properties. Kang et al. [7] use a sparse representation-based model for both filtering Rician noise and deblurring MR images. The model incorporates a regularization term to preserve small details and repeated patterns while using a non-convex total variation term for preserving edges. Li et al. [42] presented an improved dictionary learning model for medical image fusion denoising. By incorporating a low rank and sparse regularization term, into the sparse model, this proposal can remove noise while preserving textural details.

Further contributions, such as those proposed by Veraart et al. [9], use random matrix theory for filtering typical fluctuations originated from thermal noise in diffusion-weighted MR images. Phophalia and Mitra [43] use Kernel principal component analysis (KPCA) and Rough Set Theory (RST) for denoising volumetric MR data. RST is used to group similar voxels forming basis vectors, which are projected into a kernel space for performing PCA where the linear separation is possible, and the filtering is carried out.

Baselice et al. [44] introduced an approach from the complex domain under the argument that, in this domain, the filtering has the advantage of a simplified statistical model of the signal with Gaussian nature and zero mean. This method is an advantage over methods that operate in the amplitude domain where the noise model may not comply with being Gaussian.

Although recently some authors have focused on accurately estimating the noise [45] and correcting the bias produced by the aggregation of non-stationary distributed MR images samples, before trying to remove the noise [46,47], new research focused on denoising MR images by applying machine learning-based techniques. Chang et al. [6] presented an extension of the bilateral filter, which adapts to voxel intensity variations by including an intensity similarity function and automates the calculations of denoising parameters values, by extracting descriptors of texture issues, through an ensemble classifier of support vector machines and artificial neural networks. Other works use deep networks for exploring the similarity between neighboring slices in order to reduce the over-smoothing [5] and for denoising Dynamic contrast-enhanced MR images [8]. Zhang et al. [48] proposed a feed-forward denoising convolutional neural networks (DnCNNs) that took the batch normalization and residual learning architecture to denoising images, reducing the training process and improving the denoising performance. Based on the DnCNN, Jiang et al. [49] proposed a denoising method for brain MR images. They proposed a multichannel version of the DnCNN for dealing with 3D images, improving filtering performance in various noise levels. Kidoh et al. [50] also proposed a deep learning-based reconstruction method for denoising brain MR images. Their method reduces image noise while preserving image quality outperforming the DnCNN.

This paper presents a filter for denoising MR magnitude images. Unlike some of the works presented based on NLM [17,18,19,20], or SVD [26,27], or sparse representations [7,40,41,42], or some combination of these [21,28,29,30,41], we present a novel method, which does not propose a new sparse model but fully exploits one of the most typical representative examples of sparse representation, dictionary learning. We use dictionary learning for both orchestrating the non-local and the SVD approach efficiently, and for outperforming the representation of fine details of the image, by (1) enhancing the learned dictionary and determining the weighting coefficients of the patches, according to the global influence of the dictionary atoms, for filtering noise by aggregation, while avoiding blurring and keeping fine details. (2) Establishing the self-similarity of the image efficiently, in a clean space by analyzing the dictionary atoms and the sparse coding matrix, which allows us to accurately recover similar patches throughout the volume, without resorting to the exhaustive comparison of each patch in the volume, because the self-similarity can be inferred from the sparse coding matrix. Thus, we minimize the appearance of artifacts. (3) Unlike [25,26,27], which establishes complex criteria that use optimization models to determine the best low-rank approximation for filtering noise, our method, based on the similar patches identified from the sparse representation, determines simply and efficiently, the best low-rank approximation according to the estimated noise level of the image, thus reducing the remaining noise in the denoised image.

The rest of this paper is organized as follows: Section 2 presents the fundamentals of sparse representations. Section 3 presents the proposed method. Section 4 presents the results and discussion. Finally, Section 5 presents the conclusions of this work.

## 2. Sparse Representation

Dictionary Learning is a typical example of sparse representation. It aims to find the sparse representation of a set of signals Y from a dictionary D, learned from the signals themselves [51]. i.e., Y ≈ DA where A is the sparse coding matrix or the sparse representation of Y.

Formally, let $Y:=\left[y_{1}y_{2}\cdots y_{P}\right]\in R^{n\times P}$ be a matrix representing a set of P*n*-dimensional signals, the dictionary learning aims to find a dictionary $D:=\left[d_{1}d_{2}\cdots d_{K}\right]\in R^{n\times K}$ and a sparse matrix $A:=\left[\alpha_{1}\alpha_{2}\cdots \alpha_{P}\right]\in R^{K\times P}$ such that Y can be approximated by a linear combination of the basis vectors (column vectors known as atoms) of D according to A. Most of the coefficients $\alpha_{i,j}$ of A are zeros, or close to zero [52]. Dictionary learning can typically be formulated as an optimization problem, as Equation (1) indicates:

(1) $$\begin{array}{c} \min_{D,A}\sum_{c=1}^{P}{∥y_{c}-D\alpha_{c}∥}_{2}^{2}\\ s.t.\\ {∥\alpha_{c}∥}_{0}\leq L \end{array}$$

In this formulation, $y_{c}$ is the *c*-th column vector (signal) of Y, D is learned from the signals themselves, L is a regularization parameter, and $\alpha_{c}$ is the *c*-th column vector of A. The term ${∥\alpha_{c}∥}_{0}$ measures the sparsity of the decomposition. It can be understood as the number of coefficients different from zero in $\alpha_{c}$, or sparse coefficients, to approximate the signals in Y as sparse as possible.

The KSVD algorithm proposed by Aharon et al. in [53,54] allows us to obtain the optimal values of D and A in Equation (1). By analyzing the sparse representation given by D and A, some characteristics of the signals in Y can be detected. Aspects such as the similarity between the signals and their rarity, are easily manageable from their sparse representation and can be useful for improving the filtering process. This will be addressed below.

## 3. Proposed Method for MRI Denoising

The typical procedure for denoising images, via sparse representations, consist of decompose the noisy image I into full overlapped n×n patches to convert them into signals and filter them using a model, such as that in Equation (1). This procedure can be extended in the case of MR images for processing the whole volume; however, as mentioned before, noise in MR images follows a Rician distribution, which is non-additive but dependent on the data. To handle this type of noise, and to get an accurate estimation of its stabilized standard deviation γ, we apply the variance stabilization transformation (VST) proposed by Foi [12] before processing the noisy volume V, which give us $V_{\mathrm{VST}}$. Then, we divide the volume $V_{\mathrm{VST}}$ into full overlapped sub-volumes of n×n×n. Each sub-volume is converted into a signal $y_{c}\in R^{n^{3}\times 1}$ to conform the matrix $Y:=\left[y_{1}y_{2}\cdots y_{P}\right]\in R^{n^{3}\times P}$, where P is the number of signals. Now Y can be reconstructed solving Equation (1) by using the KSVD algorithm.

**Figure 1:**
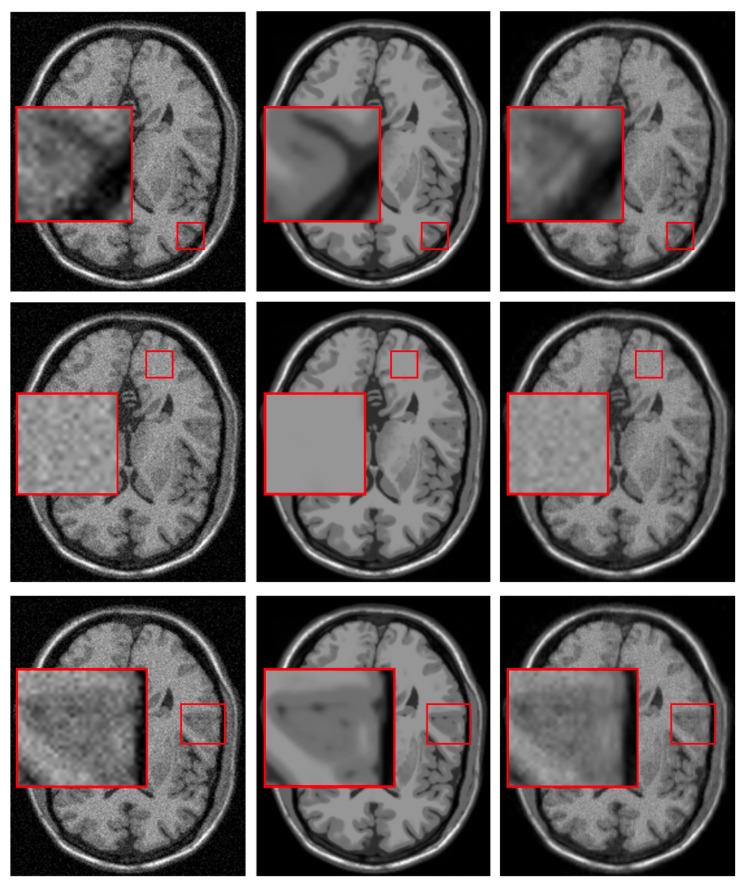
Issues of the KSVD filtering. Columns from left to right correspond to a phantom noise-free image added with 5% Rician noise, original phantom noise-free image, and image denoised by the KSVD. The third column from top to down corresponds to artifacts, residual noise, and blurring issues that the KSVD produces.

**Figure 2:**
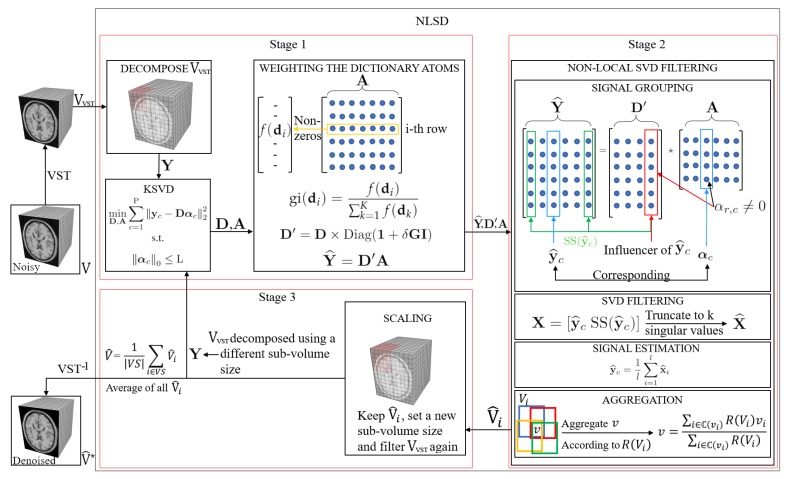
Block diagram illustrating the three steps of the proposed method.

However, the reconstruction given by DA leads to the recovery of an MR image suffering from blurring effect, artifacts, and that keeps some residual noise, as shown in Figure 1. To overcome this, we focus on three important issues of the sparse representations: the weighting of its atoms, the self-similarity that emerges from these, and the scales that these representations can handle. The three steps that make up our method address each of the issues above. Figure 2 illustrates the steps of our method.

### 3.1. Weighting the Dictionary Atoms

In digital image processing, there exist several approaches for denoising images corrupted by noise; one of them is filtering in the spatial domain using masks. The coefficients of the spatial masks are set depending on both the noise level and the type of noise. For Gaussian noise, the coefficients are weighting factors set according to the distance to the center of the mask [55]. The central coefficient gives the weight of the pixel under analysis during the denoising process. The greater this coefficient is, the more similar the filtered pixel will be to the original. This similarity implies that this coefficient can control the blurring produced by the smoothing process. We apply this reasoning to the dictionary learning, but instead of weighting the pixels of the image, we weight the atoms.

For denoising the image while keeping fine details, which is essential in MR images, this new approach proposes weighting the atoms according to its global influence. We define the global influence of a dictionary atom as the probability that such an atom appears as a linear combination in the reconstruction of a signal. i.e., For example, after finding the optimal A in Equation (1), the global influence of an atoms is calculated as the number of non-zero coefficients corresponding to that atom in A. Formally, the global influence gi of an atom $d_{c}$ of D is equal to the probability that $d_{c}$ affects a signal, as Equation (2) indicates.

(2) $$\mathrm{gi}\left(d_{c}\right)=\frac{f(d_{c})}{\sum_{k=1}^{K}f\left(d_{k}\right)}$$

(3) $$f\left(d_{c}\right)=\sum_{p=1}^{P}g\left(\alpha_{c,p}\right)$$

(4) $$g\left(\alpha_{c,p}\right)=\left\{\begin{array}{cc} 1, & \mathrm{if}\;\alpha_{c,p}\neq 0\\ 0, & \mathrm{otherwise} \end{array}\right.$$

where $\alpha_{c,p}$ is the element entry of the *c*-th row and the *p*-th column of the matrix A. The global influence is used to reinforce the effect of the more frequent atoms in the signals, to reduce the blurring caused by the average of sub-volumes during the aggregation. In the case of brain MR images, this global influence will be used for reinforcing atoms that represent common patterns found in these images, like the edges between brain structures, and therefore, for preventing the blurring in such patterns. Equation (5) updates the dictionary for reinforcing the atoms according to their global influence.

(5) $$D^{\prime }=D\times \mathrm{Diag}\left(1+\delta \mathrm{GI}\right)$$

where $\mathrm{GI}:=[\mathrm{gi}\left(d_{1}\right),\mathrm{gi}\left(d_{2}\right),\cdots ,\mathrm{gi}\left(d_{K}\right)]$ is a vector containing the global influence of all atoms, δ is a constant for controlling the influence (δ=8, which was experimentally obtained by numerical tests). 1 is a vector of ones, and Diag(·) is a K×K diagonal matrix with diagonal element entries equal to 1+δGI. Now $\hat{Y}$, a noise-free reconstruction of Y that keeps fine details, is computed using Equation (6).

(6) $$\hat{Y}=D^{\prime }A$$

Although $\hat{Y}$, improves the representation of fine details, there is still some residual noise that can be eliminated, taking advantage of the self-similarity of the image, which can be easily examined from the sparse representation given by $D^{\prime }$ and A. This will be addressed below.

### 3.2. Non-Local SVD Filtering

#### 3.2.1. Signal Grouping

Non-local filtering overcomes local filtering [56,57]; however, it is dependent on parameters such as search windows and thresholds [18]. Also, it is affected by searching for similar patches in a noisy space, which may affect the patch grouping and therefore lead to a bad estimation of the pixel. Instead, we search for similar signals in the clean space given by $D^{\prime }A$ without searching windows, because the sparse representation allows examining the self-similarity of the image through the dictionary atoms.

**Figure 3:**
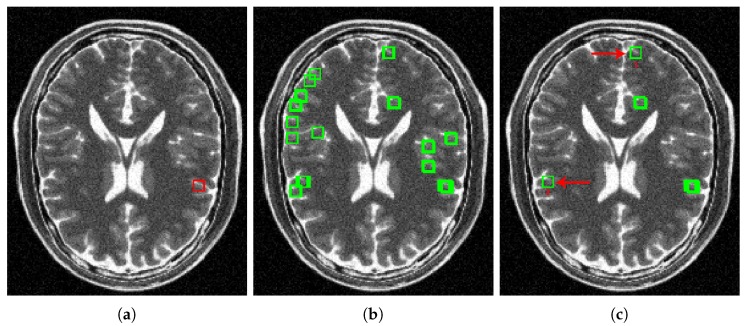

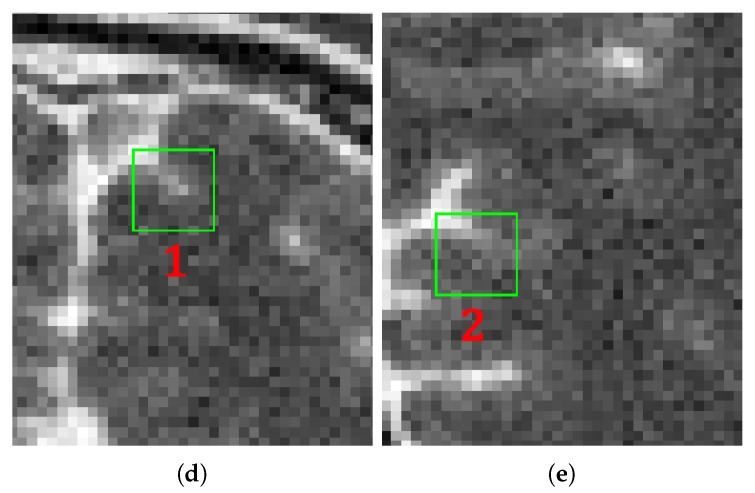
Example of a reference signal and its corresponding sets of Candidates to similar signals CSS, and Similar signals SS. (**a**) Example of a reference signal. (**b**) Candidates to similar signals of the reference signal in (**a**). (**c**) Similar signals of the reference signal in (B). (**d**) Zoom-in of the reference signal in (**a**). (**e**) Zoom-in of the similar signals 1 and 2, demonstrating that our method can locate similar signals away from the reference signal.

For performing non-local filtering based on sparse representations, we rely on the fact that similar signals admit similar sparse representations [58,59]; hence, to find similar signals to a given signal ${\hat{y}}_{c}$, we starting determining which atoms make up ${\hat{y}}_{c}$ by using Equation (7).

(7) $$I\left({\hat{y}}_{c}\right):=\left\{r:\alpha_{r,c}\neq 0,\;r=1,2,\cdots ,K\right\}$$

where K is the number of atoms in $D^{\prime }$ (number of rows of A). I denotes the set of influencer atoms of ${\hat{y}}_{c}$ i.e., the set of atoms belonging to $D^{\prime }$ that are linearly combined according to $\alpha_{c}$ for generating ${\hat{y}}_{c}$ (Figure 2 encloses an influencer atom of ${\hat{y}}_{c}$ in a red rectangle in the SIGNAL GROUPING box). Then, we search for the most influencer atoms of $\hat{Y}$ as Equation (8) indicates.

(8) $$M\left(\hat{Y}\right):=\left\{\left(r,c\right):|\alpha_{r,c}\left|=\max(\right|\alpha_{c}\left|\right),\;c=1,2,\cdots ,P\right\}$$

where $|\alpha_{c}\left|:=[\right|\alpha_{1,c}\left|,\right|\alpha_{2,c}\left|,\cdots ,\right|\alpha_{K,c}\left|\right]$ is the vector $\alpha_{c}$ with its components in absolut value, $\max(|\alpha_{c}|)$ denotes the largest component of $|\alpha_{c}|$, and P is the number of signals in $\hat{Y}$. M denotes the set of ordered pairs (r,c) meaning that $d_{r}$ of $D^{\prime }$ is the greatest influencer atom of the signal ${\hat{y}}_{c}$ of $\hat{Y}$. From the two previous sets, the set $\mathrm{CSS}({\hat{y}}_{c})$ of candidates to similar signals of ${\hat{y}}_{c}$ is defined according to Equation (9).

(9) $$\mathrm{CSS}\left({\hat{y}}_{c}\right)=\left\{{\hat{y}}_{j}:\left(r,j\right)\in M\left(\hat{Y}\right)\wedge r\in I\left({\hat{y}}_{c}\right)\right\}$$

where $\mathrm{CSS}({\hat{y}}_{c})$ contains all the signals ${\hat{y}}_{j}$ of $\hat{Y}$ whose greatest influencer atom belongs to $I({\hat{y}}_{c})$. Figure 3a shows an example of a given reference signal ${\hat{y}}_{c}$ in a MR image. Figure 3b shows the set of candidates to similar signals $\mathrm{CSS}({\hat{y}}_{c})$ from the given reference signal of Figure 3a, obtained using Equation (9). For obtaining the set of similar signals of ${\hat{y}}_{c}$ denoted by $\mathrm{SS}({\hat{y}}_{c})$ (signals pointed out by green arrows in Figure 2), and additionally, to get a better filtering performance in visual terms, we rely on the fact that the human eye is relatively less sensitive to dark signals than to bright ones [60]. Therefore, we do not define a fixed threshold for selecting similar signals, but a variable one, dependent on both the magnitude of the reference signal and the noise level of the image. This variable threshold will allow reference signals with a large magnitude admit less difference to similar signals than the dark ones; in addition, will allow increasing the number of similar signals for MR images with high noise levels to get a better estimation of the noise-free reference signal. Hence, we define $\mathrm{SS}({\hat{y}}_{c})$ according to Equation (10).

(10) $$\mathrm{SS}\left({\hat{y}}_{c}\right):=\left\{{\hat{y}}_{j}\in \mathrm{CSS}\left({\hat{y}}_{c}\right):\frac{∥{\hat{y}}_{j}-{\hat{y}}_{c}∥}{∥{\hat{y}}_{c}∥}\leq \left(b-a\frac{∥{\hat{y}}_{c}∥}{∥1_{n^{3}}∥}\right)\left(1+\gamma \right)\right\}$$

where *b* is the upper limit and *b*−*a* is the lower limit, respectively, of the interval containing the variable threshold that establishes the relative similarity between the signals (a=0.06 and b=0.18, which were experimentally obtained by numerical tests). $1_{n^{3}}$ is an $n^{3}$-dimensional vector of ones (with *n* being the edge of the sub-volume). γ is the estimated noise level. The left side of the inequality in Equation (10) is undefined when $∥{\hat{y}}_{c}∥=0$. Hence, we convert it into $∥{\hat{y}}_{j}∥$ when $∥{\hat{y}}_{c}∥\;\leq \;10^{-6}$. Please note that a bright signal (large magnitude signal) admits less difference in their similar signals than a dark one because of the magnitude of the threshold $b-a\frac{∥{\hat{y}}_{c}∥}{∥1_{n^{3}}∥}$ decreases as long as $∥{\hat{y}}_{c}∥$ increases. Also note that, although the threshold is inversely proportional to the magnitude of the reference signal in the interval [b−a,b], this threshold will be greater for signals of high noise level images than for signals of low noise level images, because of the term (1+γ). We can increase the threshold for collecting similar signals on high noise level images with out significantly affect the subsequent estimation of the noise-free reference signal, because we search for similar signals in the clean space given by the sparse representation.

Figure 3c shows the set of similar signals $\mathrm{SS}({\hat{y}}_{c})$ of the reference signal given in Figure 3a obtained after applying Equation (10) to $\mathrm{CSS}({\hat{y}}_{c})$. The red arrows in Figure 3c point out similar signals away from the reference signal, demonstrating that the sparse representation allows us to locate similar signals throughout the MR image, without examining the entire volume. It also shows that our method can locate signals in a space not restricted to search windows. Therefore we can collect a larger number of similar signals (with respect to methods that use such windows), which improves the denoising.

Now, the set $\mathrm{SS}({\hat{y}}_{c})$ can be used for estimating ${\hat{y}}_{c}$ in a non-local way; however, as was mentioned before, all the signals in $\hat{Y}$ keep some residual noise, and a better estimation could be carried out by a cleaner version of $\mathrm{SS}({\hat{y}}_{c})$.

#### 3.2.2. Efficient SVD Filtering

A known way for reducing the noise of an image is by analyzing its singular value decomposition (SVD). SVD is a matrix factorization that allows for the decomposition of a given matrix into three new matrices U, Σ and V so that $X=U\Sigma V^{⊺}$, where U and V are orthogonal matrices, and Σ is a diagonal matrix whose diagonal entries are the singular values of X. Alternatively, in summation form, $X=\sum_{i=1}^{n}s_{i}u_{i}v_{i}$, which can be understood as a weighted sum of component images, where the Eigen-images corresponding to the smallest singular values are commonly associated with noise [61]. Therefore, a vital issue for denoising based on SVD is determining what singular values correspond to the component noisy images. According to the Eckart–Young–Mirsky theorem [62], given a matrix $X=U\Sigma V^{⊺}$ then for any rank-*k* matrix $\hat{X}$, it is fulfilled that:

(11) $${∥X-\hat{X}∥}_{F}={∥X-U\Sigma_{k}V^{⊺}∥}_{F}\geq \sqrt{\sum_{i=k+1}^{n}\sigma_{i}^{2}}$$

where ${∥\cdot ∥}_{F}$ is the Frobenius norm, $\Sigma_{k}$ is the Σ matrix of the SVD decomposition of X with the singular values $\sigma_{k+1}=\sigma_{k+2}=\cdots =\sigma_{n}=0$. By squaring Equations (11) and (12) is obtained.

(12) $${∥X-\hat{X}∥}_{F}^{2}={∥X-U\Sigma_{k}V^{⊺}∥}_{F}^{2}\geq \sum_{i=k+1}^{n}\sigma_{i}^{2}$$

Taking the right side of the inequality and matching $\gamma^{2}$, where γ is the stabilized standard deviation of noise (estimated according to [12]), we have:

(13) $$\sum_{i=k+1}^{n}\sigma_{i}^{2}\geq \gamma^{2}$$

Equation (13) tells us that, when the sum of squares of the n−k less significant singular values of X is equal or greater than $\gamma^{2}$, we have the best *k* for filtering the noise of X. These n−k less significant singular values correspond to the noisy component images (noisy component volumes in our case), and therefore, using only the first *k* more significant singular values we discard such component noisy images of X.

Now, for filtering the residual noise of both ${\hat{y}}_{c}$ and $\mathrm{SS}({\hat{y}}_{c})$ let us define X as the matrix made up of ${\hat{y}}_{c}$ and its similar signals $\mathrm{SS}({\hat{y}}_{c})$, i.e., $X=\left[{\hat{y}}_{c}\;\mathrm{SS}\left({\hat{y}}_{c}\right)\right]$, and following Equation (13) the noise-free version $\hat{X}$ is obtained with the low rank approximation given by $U\Sigma_{k}V^{⊺}$ considering the estimated level of noise γ.

#### 3.2.3. Signal Estimation

${\hat{y}}_{c}$ is updated from $\hat{X}$, according to Equation (14).

(14) $${\hat{y}}_{c}=\frac{1}{l}\sum_{i=1}^{l}{\hat{x}}_{i}$$

where ${\hat{x}}_{i}$ is the i-th signal of $\hat{X}$ and *l* is the number of signals in $\hat{X}$.

#### 3.2.4. Aggregation

The restoration of the MR sub-volumes from $\hat{Y}$ involves the aggregation of overlapped voxels, which can lead to the blurry recovery of the voxel. However, as mentioned before, weighting the voxels can control blurring. Now, the voxels to be aggregated belong to sub-volumes (signals), which may be frequent (has many similar signals) or rare (has a few similar signals). Since the frequent sub-volumes are better estimated than the rare sub-volumes because of the number of signals averaged in Equation (14), these voxels should have more weight than the voxels from the rare sub-volumes. Therefore, we propose weighting the voxel according to the rarity of the signal they belong to.

Similar to the procedure outlined in Leal et al. [63], the global influence of atoms can be used to establish the rarity of a signal. For example, a signal will be rare if it accumulates little global influence; it will be frequent if it accumulates a lot of global influence. Equation (15) allows computing the rarity of a signal based on the global influence of the atoms that make it up.

(15) $$R\left({\hat{y}}_{c}\right)=\langle \mathrm{GI},\alpha_{c}\rangle$$

where $R({\hat{y}}_{c})$ denotes the rarity score of the signal ${\hat{y}}_{c}$, 〈,〉 denotes the dot product, and $\alpha_{c}$ is the *c*-th column vector of A, which represents the sparse representation of ${\hat{y}}_{c}$ on $D^{\prime }$.

High scores of Equation (15) are related to frequent signals, while low scores are related to rare signals. The denoised version of the voxel *v* is obtained according to Equation (16), after converting back the signals into sub-volumes.

(16) $$v=\frac{\sum_{i\in C(v_{i})}R\left(V_{i}\right)v_{i}}{\sum_{i\in C(v_{i})}R\left(V_{i}\right)}$$

where $v_{i}$ is a version of the voxel *v* contained in the sub-volume $V_{i}$, $R(V_{i})$ is the rarity score of the signal ${\hat{y}}_{i}$ corresponding to the sub-volume $V_{i}$, and $C(v_{i})$ denotes the set of sub-volumes $V_{i}$ that contains a version of *v*.

### 3.3. Scaling

An important parameter for denoising based on sparse representations is the patch size. Large patches lead to denoised images suffering from blurring effect, while small patches lead to denoised images that can keep a high level of noise. However, small patches keep also fine details of the original image.

Averaging images denoised using different patch sizes can carry out a good trade-off between blurring and fine details. The noisy image can be denoised several times; but, each time with a different patch size, then the denoised versions of the image are averaged. The different patch sizes for processing the image are known as scales. Let us define ${\hat{V}}_{n}$ as the denoised version of a noisy MR image *V* using the proposed method with a sub-volume of size n×n×n. The averaged denoised version $\hat{V}$ of *V*, is obtained using Equation (17).

(17) $$\hat{V}=\frac{1}{\left|VS\right|}\sum_{i\in VS}{\hat{V}}_{i}$$

where |VS| denotes the number of elements in VS, and VS is the set of different sub-volume sizes for processing *V*. In our tests, we achieved the best results by averaging the denoised versions ${\hat{V}}_{3}$ and ${\hat{V}}_{4}$. To obtain the final output ${\hat{V}}^{*}$, we apply the inverse VST (VST^−1^) to the denoised volume $\hat{V}$. The denoising of Rician data using the proposed method, Non-local SVD Denoising—NLSD, is synthesized by Equation (18)

(18) $${\hat{V}}^{*}=VST^{-1}\left(NLSD\left(VST\left(V,\sigma \right),\gamma \right),\sigma \right)$$

where γ is the stabilized standard deviation induced by the VST, V is the volume corrupted by Rician noise with standard deviation σ.

## 4. Results and Discussion

We conducted several experiments to obtain the values of the parameters for the optimal setting of our method and to compare some state-of-the-art methods. These experiments were conducted in MATLAB R2016a running under the Windows 10 Professional OS on an Intel Core i7 First Generation CPU with 8GB of memory.

A dataset of MR phantom images (1 T1w, 1 T2w, and 1 PDw) from the BrainWeb data base [64], with a size of 181×217×181 voxels (resolution = 1 mm^3^), was used for numerical comparisons, as well as for setting the optimal parameter values of our method.

### 4.1. Parameter Estimation

The first stage of our method, which is preceded by the VST, aims to compute the global influence to reinforce the atoms for avoiding blurring in the denoised image. This reinforcing is controlled by the parameter δ, which does not intervene in the remaining stages. For determining a suitable value for this parameter, it was performed an experiment which consisted of corrupting the dataset of MR phantom images with different levels of Rician noise (1–10% of the maximum intensity), i.e., from each volume of the dataset were obtained 10 different noisy versions, one per each level of noise, for a total of 30 corrupted images. Then, we ran the first stage of our method over each corrupted image, followed by the inverse VST. Finally, we calculate the widely recognized quality measures Peak Signal to Noise Ratio (PSNR), and the Structural Similarity Index (SSIM) [65] of each denoised image to establish the best value for this parameter numerically.

**Figure 4:**
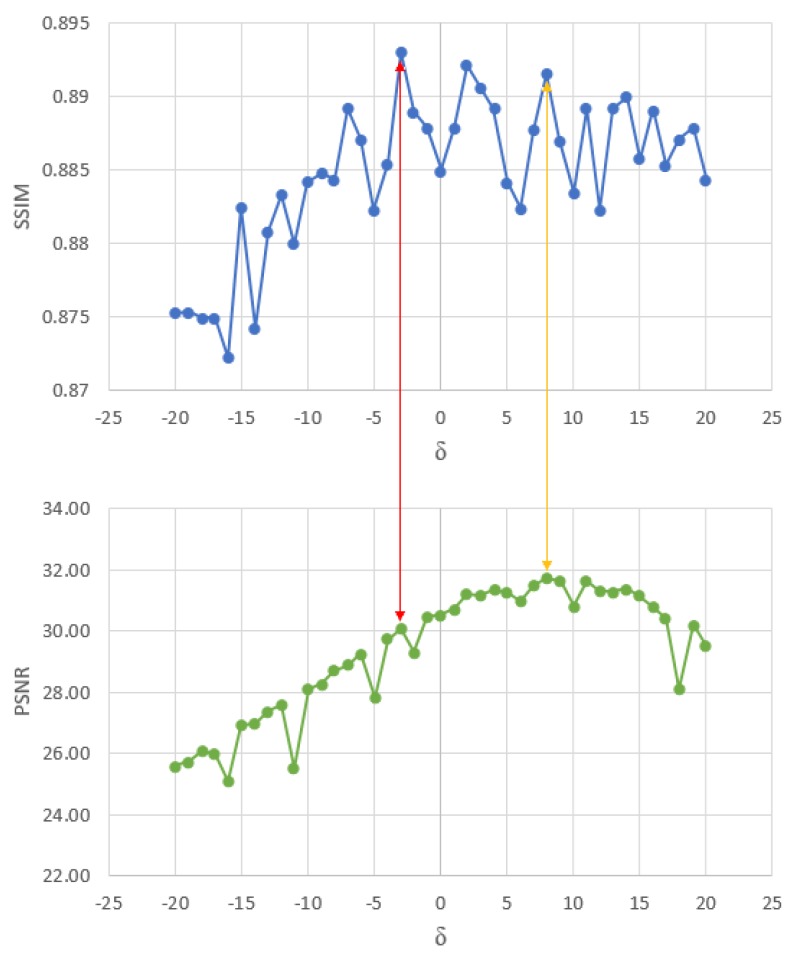
Summary of the tests performed to numerically establish the best value of the parameter δ for controlling the global influence of the atoms. The red arrow points out the maximum SSIM (blue curve) and its corresponding PSNR (green curve). The yellow arrow points out the maximum PSNR and its corresponding SSIM.

Figure 4 shows the results of applying the first stage of our method over the dataset of MR phantom images corrupted with the different Rician noise levels. Each point on the green curve is the average PSNR of the denoised images for a given value of δ, and each point on the blue curve is the average SSIM.

We observed that the PSNR is maximum (31.991) at δ=8 and the SSIM is maximum (0.8934) at δ=−3. It is preferable to choose the value of δ that generates the maximum PSNR instead of the value that generates the maximum SSIM. Since this metric (PSNR) is more sensitive to noise than the SSIM (which is more correlated with a human visual system) [66], and by choosing the value of δ that give us the maximum PSNR we are choosing the cleaner search space for the next stage. Additionally, the best PSNR and SSIM pair is reached at δ=8.

**Figure 5:**
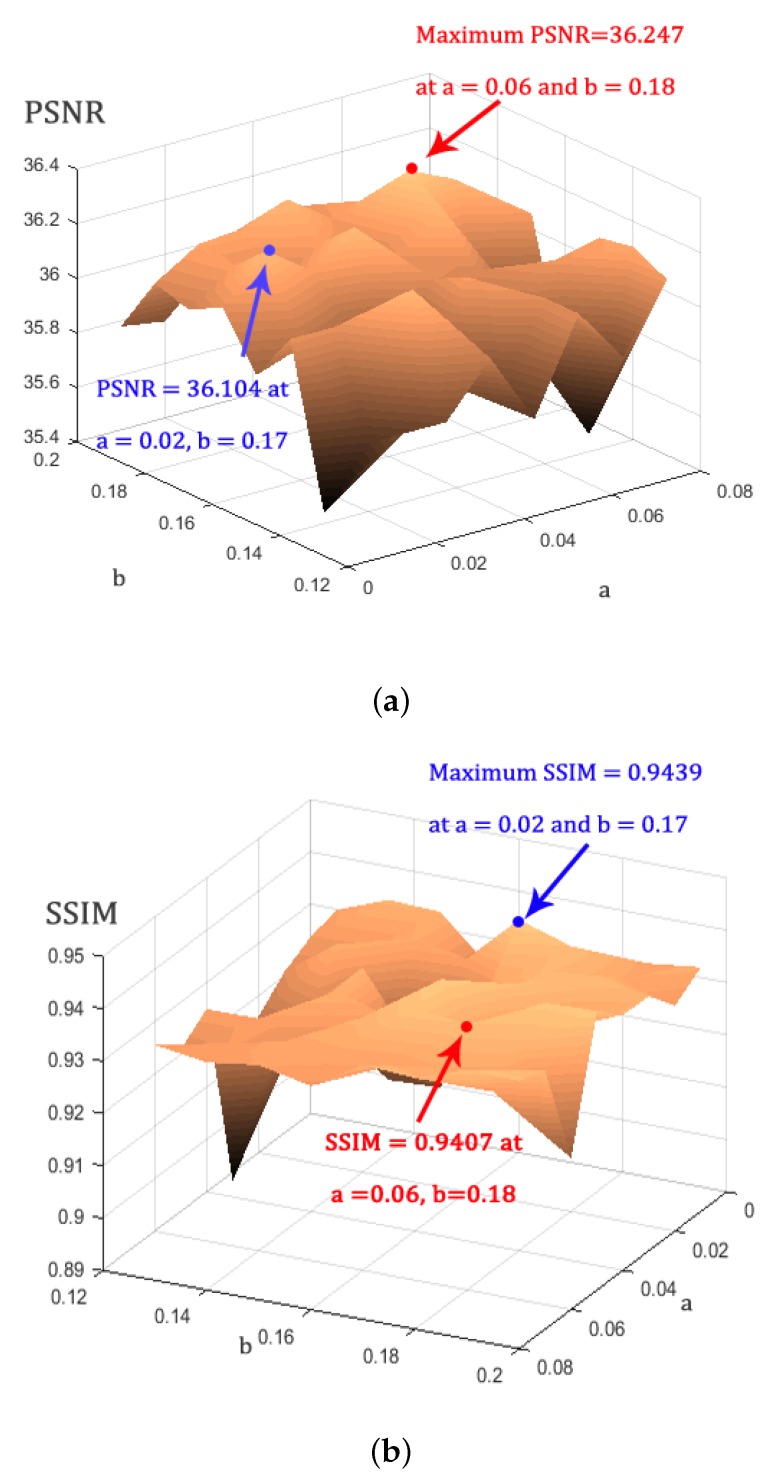
Summary of the tests performed to numerically establish the best values for the parameters *a* and *b* for controlling the similarity between the signals according to the magnitude of the reference signal and the estimated level of noise. (**a**) PSNR surface where each point corresponds to the average PSNR of the MR phantom images corruped with different Rician noise levels and denoised using the two first stages of our method. (**b**) SSIM surface obtained similarly to the PSNR surface.

The second stage of our method is to perform the Non-Local-SVD filtering. At this stage, we search for similar signals to a reference signal in the clean space given by the first stage. We defined a variable threshold in the interval [b−a,b] for establishing the level of similarity between the signals (right side of the inequality in Equation (10)). This threshold depends on the magnitude of ${\hat{y}}_{c}$ and the noise level γ estimated in the first stage. The interval where the threshold moves is determined by the constants *a* and *b*. To establish a suitable value for these constants, it was performed an experiment which consisted of running the second stage of our method over the sparse representation $\hat{Y}=D^{\prime }A$ of each denoised image produced by the tests of the first stage (previous to the application of the inverse VST), trying different values for *a* and *b*. In this experiment, the application of the second stage of our method was followed by the inverse VST. Figure 5 shows the results. Each point on the PSNR surface (Figure 5a) represents the average of the PSNRs computed after applying the second stage or our method over the images denoised by the first stage, for a given pair of values *a* and *b*. Similarly, Each point on the SSIM surface (Figure 5b) is the average SSIM.

As shown in Figure 5a, the maximum PSNR is equal to 36.247, which is reached at a=0.06 and b=0.18. At this point, the SSIM is 0.9407. As shown in Figure 5b, the maximum SSIM is 0.9439, which is reached at a=0.02 and b=0.17. At this point, the PSNR is 36.104. We chose the pair a=0.06 and b=0.18 because the increment of the PSNR at this point with respect to the PSNR at the point where the maximum SSIM is reached, is more significant than the increment of the SSIM at a=0.02 and b=0.17, with respect to the SSIM at the point where the maximum PSNR is reached.

### 4.2. Methods Comparison

We compared the proposed method for MRI denoising, NLSD, to the following widely adopted filters: KSVD [53], PRINLM [21] (implementation downloaded from [67]), BM4D [32] (implementation downloaded from [68]), and DnCNN [48] (implementation dowloaded from [69]). The implementation of the methods PRINLM and BM4D used for comparison are developed in MATLAB with its core developed in C++. The DnCNN, the KSVD, and the NLSD, are implemented purely in MATLAB and do not use any graphic acceleration. The application of the KSVD was preceded by the VST, and followed by its inverse. We performed both quantitative and qualitative comparisons for demonstrating the effectivity or our method.

#### 4.2.1. Synthetic Data

For numerical comparisons, we used the MR phantom images dataset, corrupted with Rician noise (1–10% of the maximum intensity). The PSNR, and the SSIM were used to evaluate the performance. Figure 6, Figure 7, Figure 8, Figure 9, Figure 10, Figure 11, Figure 12, Figure 13, Figure 14, Figure 15, Figure 16 and Figure 17 and Table 1, Table 2, Table 3 and Table 4 show the results of these comparisons.

**Figure 6:**
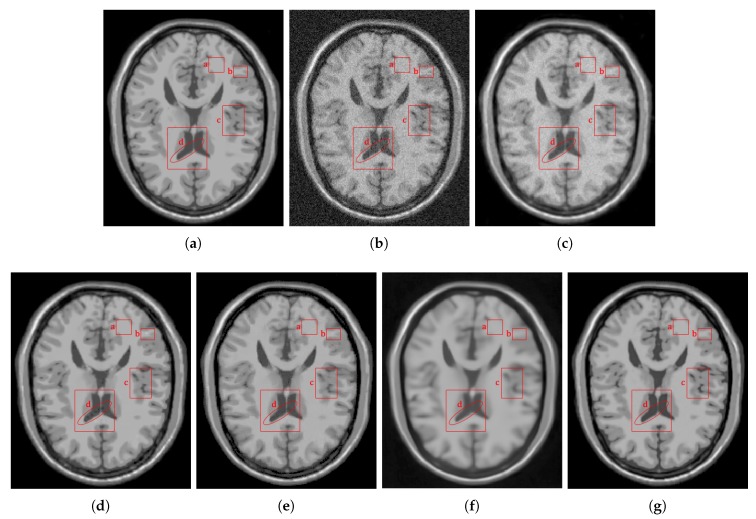
Comparison of the denoising methods on the T1w synthetic noise-free MR image corrupted with 5% Rician noise. (**a**) Original synthetic noise-free MR image. (**b**) Original image corrupted with 5% Rician noise. (**c**–**g**) Denoising results of the KSVD, PRINLM, BM4D, DnCNN, and the proposed method NLSD.

**Figure 7:**
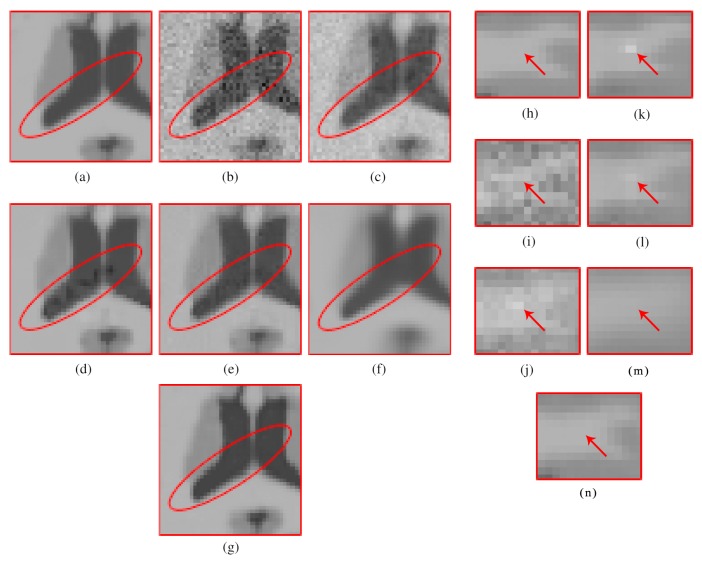
Zoom-in on the details of the regions labeled as “d” and “b” in Figure 6. Figure (**a**–**g**) show the detail labeled as “d” in Figure 6a–g respectively. Figure (**c**–**g**) Correspond to the methods KSVD, PRINLM, BM4D, DnCNN and NLSD, respectively. Figure (**h**–**n**) show the detail labeled as “b” in Figure 6a–g respectively. Figure (**j**–**n**) Correspond to the methods KSVD, PRINLM, BM4D, DnCNN, and NLSD, respectively.

**Figure 8:**
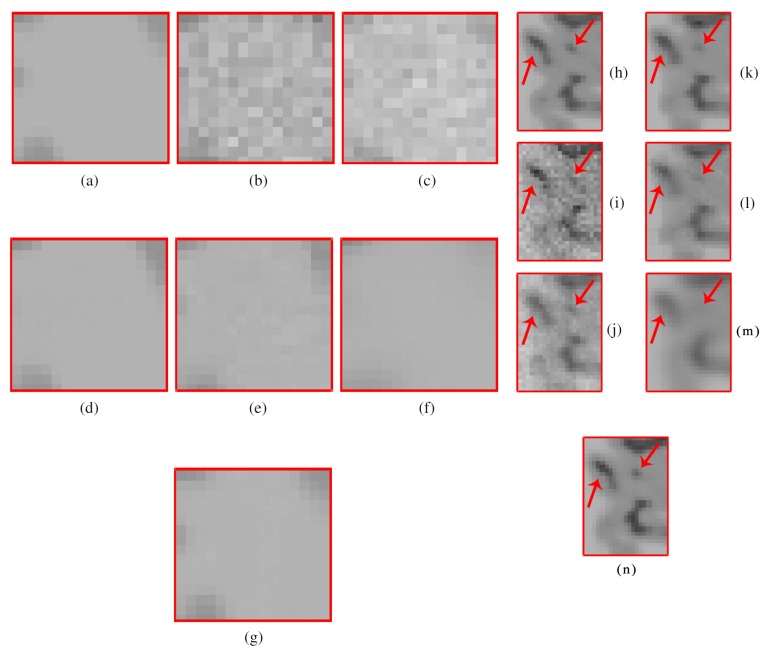
Zoom-in on the details of the regions labeled as “a” and “c” in Figure 6. Figure (**a**–**g**) correspond to the regions labeled as “a” and Figure (**h**–**n**) correspond to the regions labeled as “c”. Figure (**c**–**g**) and (**j**–**n**) correspond to the zoom-in on the results of the methods KSVD, PRINLM, BM4D, DnCNN and NLSD, respectively.

Figure 6 shows the results of the comparisons on the T1w synthetic noise-free MR image corrupted with 5% Rician noise. It shows that the methods PRINLM, BM4D, DnCNN, and NLSD perform very well removing noise; however, the PRINLM produces some artifacts as the zoom-in in Figure 7d,k show. The KSVD and the BM4D also produce artifacts but to a lesser extent, as shown in Figure 7c,e, respectively, and Figure 7j,l respectively. The DnCNN over-smooth the image and therefore fine details as shown in Figure 8m. Instead, the NLSD removes noise while keeping fine details, in addition, does not produce artifacts. The KSVD maintains a lot of residual noise, as well as the BM4D, although to a much lesser extent, as shown in Figure 8c,e, respectively. Figure 8d,g show that the PRINLM and the NLSD, respectively, do not keep residual noise. Figure 8h–n show the zoom-in on the details labeled as “c” in Figure 6. It shows that the methods KSVD, BM4D, and DnCNN are affected by the blurring effect (Figure 8j,l,m, respectively). The PRINLM (Figure 8k) and the NLSD (Figure 8n) keep fine details; however, the PRINLM tends to swell such details. Table 1 summarizes the results of the numerical tests in the T1w synthetic MR image. Bold highlights indicate the best results.

**Figure 9:**
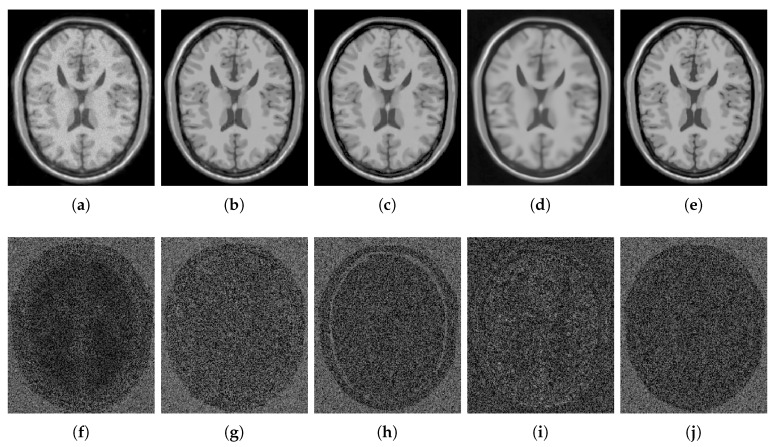
Residuals of the different results produced by the application of the methods evaluated on the noise-free T1w synthetic MR image of Figure 6a corrupted with 5% Rician noise. (**a**–**e**) are the denoising results of the methods KSVD, PRINLM, BM4D, DnCNN, and NLSD, respectively. (**f**–**j**) are the corresponding residuals.

Figure 9 qualitatively compares the denoising methods from the residual images (images resulting from the difference between the denoised and noisy images). It shows that the KSVD retains some structural information, and the DnCNN to a lesser extent, while the PRINLM, the BM4D, and the NLSD perform very well. However, the NLSD presents both a higher PSNR and a higher SSIM, as Table 1 shows.

**Figure 10:**
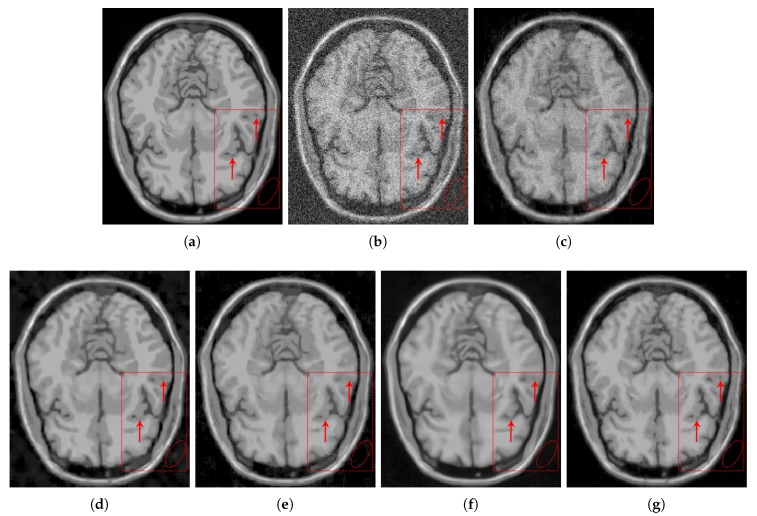
Comparison of the denoising methods on the T1w synthetic noise-free MR image corrupted with 9% Rician noise. (**a**) Original synthetic noise-free MR image. (**b**) Original image corrupted with 9% Rician noise. (**c**–**g**) Denoising results of the KSVD, PRINLM, BM4D, DnCNN and the proposed method NLSD.

**Figure 11:**
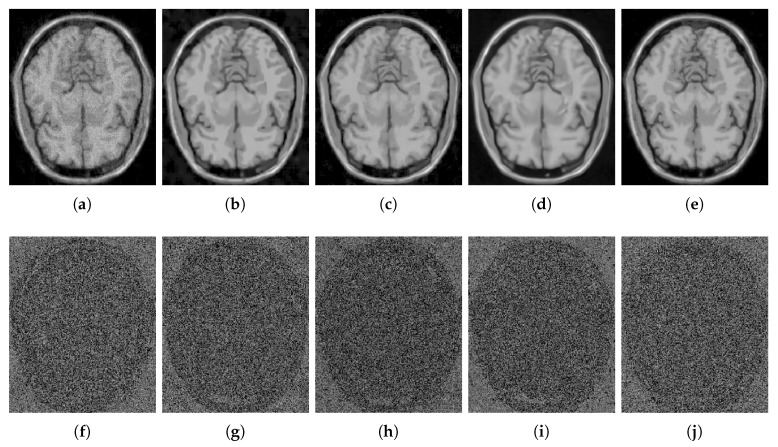
Residuals of the different results produced by the application of the methods evaluated on the noise-free T1w synthetic MR image of Figure 10a corrupted with 9% Rician noise. (**a**–**e**) are the denoising results of the methods KSVD, PRINLM, BM4D, DnCNN and NLSD, respectively. (**f**–**j**) are the corresponding residuals.

Figure 10 shows the results of the comparisons on the T1w synthetic noise-free MR image corrupted with 9% Rician noise. It shows that the PRINLM do not eliminate the noise in dark regions but tends to flatten (circled area) and destroy fine details (red arrows). BM4D also keep noise in dark areas (circled area) and also destroy fine details. The DnCNN remove noise but over-blur the image destroying fine details. The KSVD keep a lot of noise and destroy fine details. The NLSD instead keep fine details and perform very well removing noise. You can zooming in the images to appreciate the details. Figure 11 shows the qualitative comparison of the method evaluated from the residual images. No structural information is observed on the residuals of the NLSD. The residuals of the KSVD and the DnCNN present some structural information. The residuals of the PRINLM and the BM4D also present structural information to a lesser extent.

**Figure 12:**
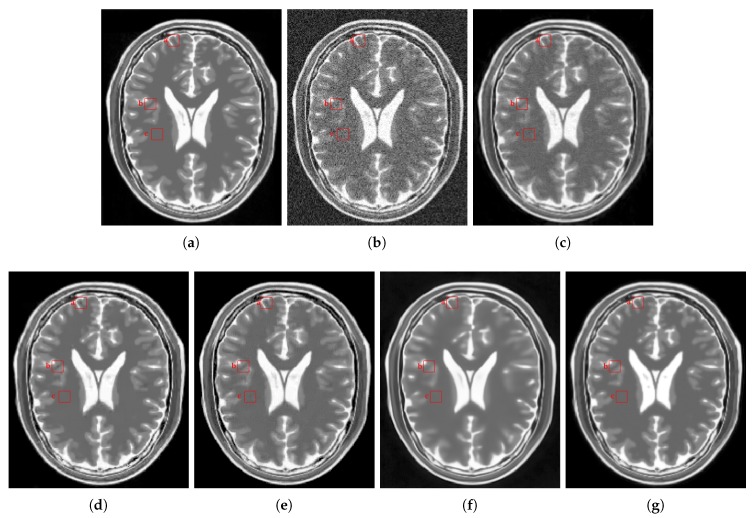
Comparison of the denoising methods on the T2w synthetic noise-free MR image corrupted with 7% Rician noise. (**a**) Original synthetic noise-free MR image. (**b**) Original image corrupted with 7% Rician noise. (**c**–**g**) Denoising results of the KSVD, the PRINLM, the BM4D, the DnCNN and the NLSD respectively.

**Figure 13:**
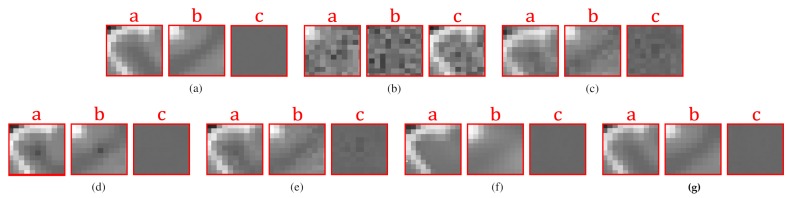
Zoom-in on the details labeled as “a”, “b”, and “c” in Figure 12. Figures (**a**–**g**) correspond to the zoom-in of the Original image, the Noisy image, the KSVD, the PRINLM, the BM4D, the DnCNN, and the NLSD, respectively.

**Figure 14:**
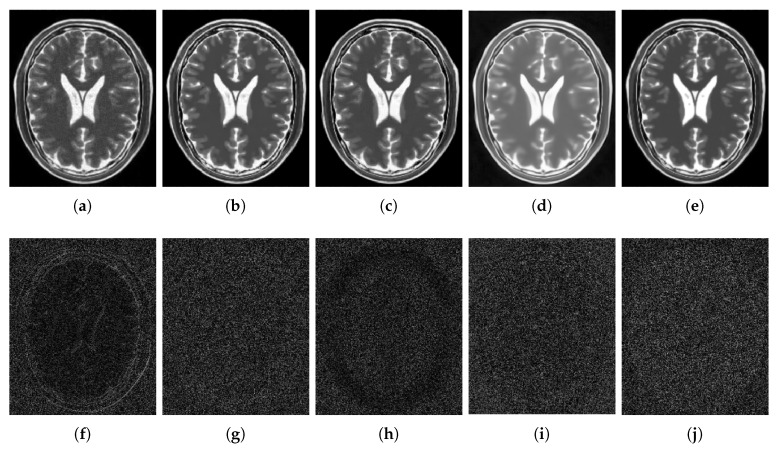
Residuals of the different results produced by the application of the methods evaluated on the noise-free T2w synthetic MR image of Figure 12a corrupted with 7% Rician noise. (**a**–**e**) are the denoising results of the methods KSVD, PRINLM, BM4D, DnCNN, and NLSD, respectively. (**f**–**j**) are the corresponding residuals.

Figure 12 shows the results of the comparisons on the T2w synthetic noise-free MR image corrupted with 7% Rician noise. It shows that the image filtered by the KSVD (Figure 12c) keeps high levels of noise, which is corroborated by the corresponding zoom-in of region “c” in Figure 13c. The BM4D also keeps noise, although to a lesser extent, as shown in region “c” in Figure 13e. The DnCNN over-blur the image and destroy fine details, as can be seen in the corresponding zoom-in of region “b” in Figure 13f. The methods PRINLM and NLSD perform very well at removing noise; however, as shown in the zoom-in of the regions “a” and “b” in Figure 13d, the PRINLM produces artifacts. The NLSD does not produce artifacts. Figure 14 qualitatively compares the denoising methods from the residual images. It shows that the KSVD presents a correlated residual image and also the BM4D. The DnCNN present a residual image that keep some estructural information, but to a lesser extent, while the PRINLM, and the NLSD perform very well. However, the NLSD presents both a higher PSNR and a higher SSIM, as Table 2 shows. Table 2 summarizes the results of the numerical tests in the T2w synthetic MR image. Bold highlights indicate the best results.

**Figure 15:**
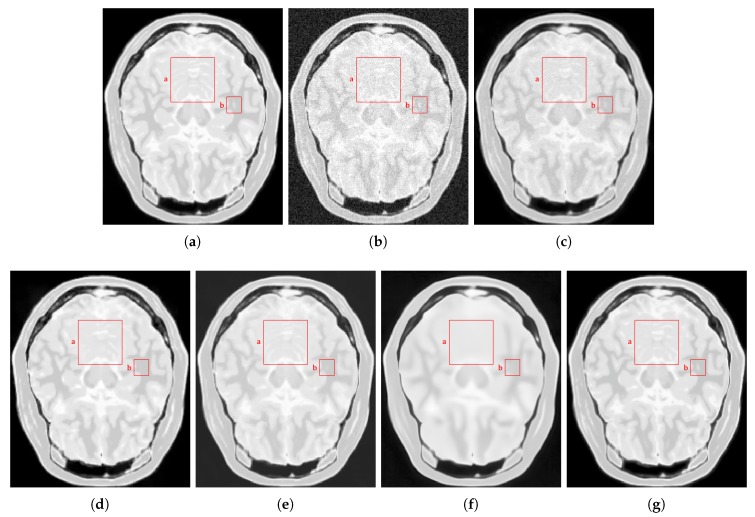
Comparison of the denoising methods on the PDw synthetic noise-free MR image corrupted with 5% Rician noise. (**a**) Original synthetic noise-free MR image. (**b**) Original image corrupted with 5% Rician noise. (**c**–**g**) are the denoising results of the KSVD, the PRINLM, the BM4D, the DnCNN and the NLSD, respectively.

**Figure 16:**
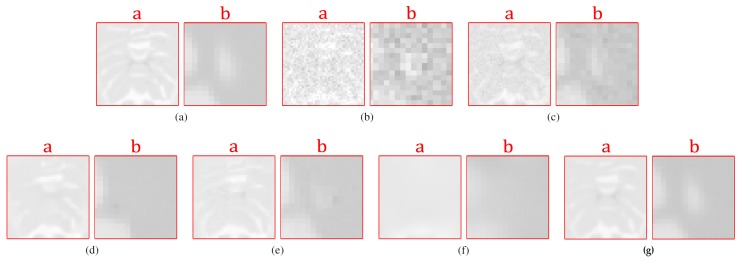
Zoom-in on the details labeled as “a” and “b” in Figure 15. Figures (**a**–**g**) correspond to the zoom-in of the Original image, the Noisy image, the KSVD, the PRINLM, the BM4D, the DnCNN and the NLSD in Figure 15, respectively.

**Figure 17:**
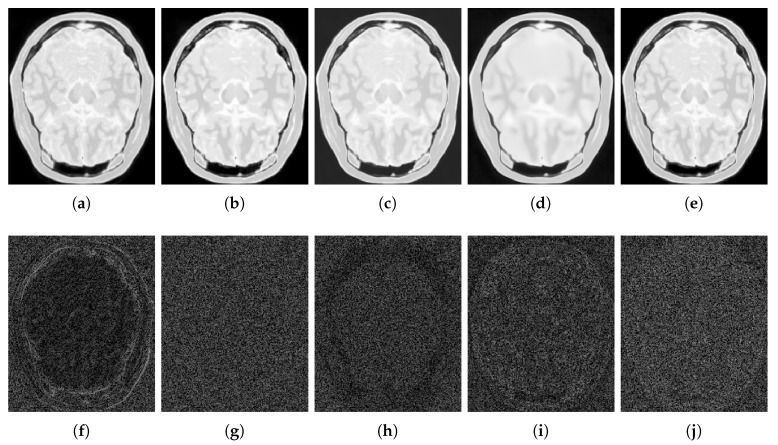
Residuals of the different results produced by the application of the methods evaluated on the noise-free PDw synthetic MR image of Figure 15a corrupted with 5% Rician noise. (**a**–**e**) are the denoising results of the methods KSVD, PRINLM, BM4D, DNCNN and NLSD, respectively. (**f**–**j**) are the corresponding residuals.

Figure 15 shows the results of the comparisons on the PDw synthetic noise-free MR image corrupted with 5% Rician noise. Figure 16 shows the zoom-in of the regions labeled as “a” and “b” in Figure 15. It shows that the BM4D and mainly the KSVD retain noise, as shown in Figure 16c,e in the corresponding zoom-in. The DnCNN perform very well filtering noise but over-blur the image destroying fine details, as can be seen in Figure 16f. The PRINLM and the NLSD perform very well, although the NLSD (Figure 16g) tends to preserve some fine structures better than the PRINLM. Figure 16d shows how the PRINLM tends to thicken or destroy such structures. Figure 17 qualitatively compares the denoising methods from the residual images. It shows that the KSVD presents a correlated residual image. The BM4D and DnCNN also shows a bit of correlation, while the PRINLM and the NLSD perform very well. However, the NLSD presents both a higher PSNR and a higher SSIM, as Table 3 shows.

Table 3 summarizes the results of the numerical tests in the PDw synthetic MR image. Bold highlights indicate the best results. Table 4 presents the running time of the methods.

#### 4.2.2. Real Data

**Figure 18:**
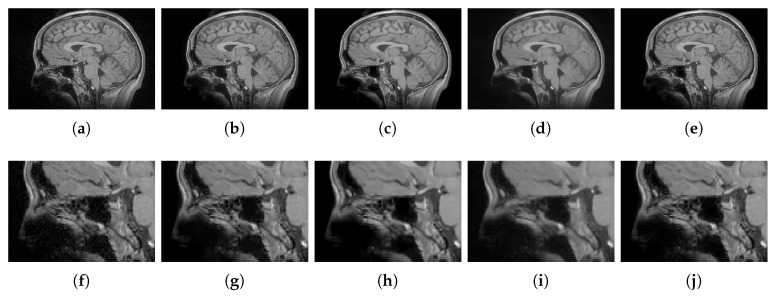
Quality comparisons on a real T1w MR image. (**a**) Original MR image, (**b**–**e**) denoising results of the PRINLM, the BM4D, DnCNN, and the NLSD. (**f**–**j**) the corresponding zoom-in of figures (**a**–**e**).

For qualitative comparisons on real clinical data, we used a data set of T1w brain MR images acquired in a 1.5T Siemens Magnetom equipped with a standard head coil. The parameters of the T1w images were: TR = 7.9 ms, TE = 3.8 ms, ACQ matrix 220×220 pixels, voxel size 0.5 mm × 0.5 mm × 0.5 mm. We compared the NLSD results to the PRINLM, the BM4D and the DnCNN results. The KSVD was not considered because its performance was much lower in the tests on synthetic data. Figure 18 shows the results. The four methods perform very well, as can be seen in their corresponding zoom-ins; however, the BM4D and DnCNN results are slightly blurry and retain some noise. The PRINLM also keeps some residual noise. It can be noted that, close to the dark areas the BM4D and the PRINLM maintain residual noise slightly flattened, as can be seen in the zoom-in. The NLSD instead reduces noise while keeping fine details and yielding better visual results. This can be seen around the skull, where almost there is no any noisy pixel. This is because of the variable threshold in Equation (10) and the better estimation of the signals achieved by the search of similar signals in the clean space given by $D^{\prime }A$.

## 5. Conclusions

In this paper, we presented a new method for MR images denoising based on non-local means, singular value decomposition, and sparse representation. Both qualitative and quantitative results validate the effectiveness of the proposed method. We demonstrated how the global influence of the dictionary atoms could act as weighting factors to improve the noise filtering while keeping the fine details of the image and reducing the blurring effect caused by the average of overlapping patches.

We also demonstrated how the strategy of non-local means could be applied directly from the dictionary without resorting to the use of search windows, which limit the search for similar signals (patches or sub-volumes) to a given region. The dictionary, instead, allows finding similar signals in any location of the MR image without exploring the whole volume; and therefore, improving the filtering. Additionally, the strategy of searching for similar signals in the clean space given by the sparse representation allows the process of collecting such signals to improve; since, the level of noise in this space is lower than in the original space given by the noise MR image. Finally, we also showed how the estimated level of noise in the image could be used for computing efficiently the number of singular values, to obtain a low-rank approximation of the set of similar signals, and to reduce their noise before proceeding to the estimation of the noise-free signal. 

## Figures and Tables

**Table 1:** Comparison of the different methods evaluated on the T1w synthetic noise-free MR image corrupted with different levels of Rician noise. Bold highlights indicate the best results.

	1%		3%		5%		7%		9%	
Method	PSNR	SSIM	PSNR	SSIM	PSNR	SSIM	PSNR	SSIM	PSNR	SSIM
Noisy	39.980	0.9658	30.451	0.8084	25.982	0.6644	23.045	0.5536	20.888	0.4693
KSVD	40.421	0.9773	32.637	0.9225	29.509	0.8605	27.588	0.8014	26.423	0.7368
PRINLM	44.299	0.9930	38.589	0.9655	35.512	0.9281	33.642	0.8654	**33.177**	0.8332
BM4D	44.688	0.9911	38.129	0.9799	35.618	0.9643	32.799	0.9404	30.412	0.9115
DnCNN	43.109	0.9756	37.843	0.9602	34.790	0.9581	33.744	0.9478	31.501	0.9163
NLSD	**44.901**	**0.9970**	**38.723**	**0.9895**	**35.801**	**0.9776**	**33.982**	**0.9630**	33.130	**0.9296**

**Table 2:** Comparison of the different methods evaluated on the T2w synthetic noise-free MR image corrupted with different levels of Rician noise. Bold highlights indicate the best results.

	1%		3%		5%		7%		9%	
Method	PSNR	SSIM	PSNR	SSIM	PSNR	SSIM	PSNR	SSIM	PSNR	SSIM
Noisy	39.992	0.9709	30.418	0.8330	26.016	0.7151	23.073	0.6242	20.928	0.5606
KSVD	40.104	0.9706	32.098	0.9166	29.342	0.8669	26.530	0.8134	24.664	0.7633
PRINLM	43.985	0.9943	37.786	0.9703	34.738	0.9262	32.918	0.8852	31.347	0.8286
BM4D	43.655	0.9614	37.166	0.9473	34.140	0.9373	31.887	0.9273	30.030	0.9100
DnCNN	42.897	0.9569	37.003	0.9354	34.362	0.9210	32.544	0.9162	31.045	0.9139
NLSD	**44.099**	**0.9978**	**37.973**	**0.9885**	**34.910**	**0.9522**	**33.546**	**0.9327**	**31.601**	**0.9233**

**Table 3:** Comparison of the different methods evaluated on the PDw synthetic noise-free MR image corrupted with different levels of Rician noise. Bold highlights indicate the best results.

	1%		3%		5%		7%		9%	
Method	PSNR	SSIM	PSNR	SSIM	PSNR	SSIM	PSNR	SSIM	PSNR	SSIM
Noisy	39.971	0.9636	30.441	0.7901	25.984	0.6341	23.088	0.5250	20.898	0.4408
KSVD	39.980	0.9639	29.633	0.8956	27.626	0.8128	26.224	0.7357	24.952	0.6589
PRINLM	44.699	0.9940	38.079	0.9752	35.111	0.9228	33.101	0.8638	32.562	0.8211
BM4D	44.603	0.9626	38.621	0.9399	35.683	0.9280	**33.653**	0.9147	31.887	0.8990
DnCNN	43.245	0.9567	37.991	0.9268	35.700	0.9372	33.419	0.9075	30.643	0.8986
NLSD	**44.980**	**0.9987**	**38.906**	**0.9894**	**35.973**	**0.9532**	33.625	**0.9387**	**32.656**	**0.9102**

**Table 4:** Implementation language and computation time of the methods evaluated.

	PRINLM	BM4D	DnCNN	KSVD	NLSD
Language	MATLAB - C++	MATLAB - C++	MATLAB	MATLAB	MATLAB
Average running time	45.32 s	46.83 s	65.64 min	70.13 min	120.84 min
